# Endocannabinoids, Related Compounds and Their Metabolic Routes

**DOI:** 10.3390/molecules191117078

**Published:** 2014-10-24

**Authors:** Filomena Fezza, Monica Bari, Rita Florio, Emanuela Talamonti, Monica Feole, Mauro Maccarrone

**Affiliations:** 1 Department of Experimental Medicine & Surgery, Tor Vergata University of Rome, 00133 Rome, Italy; E-Mails: bari@med.uniroma2.it (M.B.); monica.feole@hotmail.it (M.F.); 2 European Center for Brain Research/IRCCS Santa Lucia Foundation, 00143 Rome, Italy; 3 Department of Movement, Human and Health Sciences, Foro Italico University of Rome, 00128 Rome, Italy; E-Mail: rit.flo@libero.it; 4 Endocannabinoid Research Group, Istituto di Chimica Biomolecolare, Consiglio Nazionale delle Ricerche, 80078 Pozzuoli (NA), Italy; E-Mail: emanuelatalamonti.86@libero.it; 5 Center of Integrated Research, Campus Bio-Medico University of Rome, 00135 Rome, Italy

**Keywords:** anandamide, 2-arachidonoylglycerol, endocannabinoid metabolic routes, fatty acids

## Abstract

Endocannabinoids are lipid mediators able to bind to and activate cannabinoid receptors, the primary molecular targets responsible for the pharmacological effects of the Δ^9^-tetrahydrocannabinol. These bioactive lipids belong mainly to two classes of compounds: *N*-acylethanolamines and acylesters, being *N*-arachidonoylethanolamine (AEA) and 2-arachidonoylglycerol (2-AG), respectively, their main representatives. During the last twenty years, an ever growing number of fatty acid derivatives (endocannabinoids and endocannabinoid-like compounds) have been discovered and their activities biological is the subject of intense investigations. Here, the most recent advances, from a therapeutic point of view, on endocannabinoids, related compounds, and their metabolic routes will be reviewed.

## 1. Introduction

The biological effects of marijuana and Δ^9^-tetrahydrocannabinol (THC), its major psychoactive component, are mediated by two G protein-coupled receptors (GPCR), type-1 (CB_1_R) and type-2 (CB_2_R) cannabinoid receptors [1].

The finding of the genes that encode CBRs has led to the quest to discover their endogenous ligands [termed “endocannabinoids (eCBs)”], and the enzymes responsible for eCBs synthesis and degradation, leading in turn to the discovery of an entirely new endogenous signalling system, now known as the “endocannabinoid system (ECS)” [2,3].

**Figure 1:**
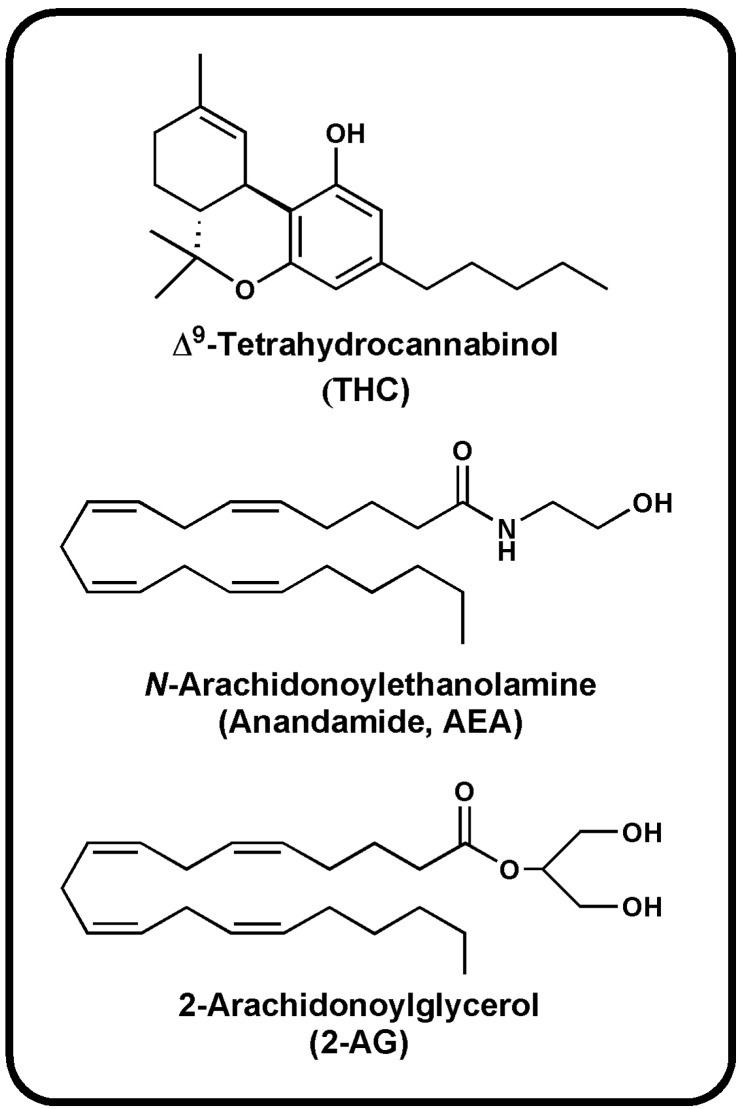
Chemical structures of THC and two prominent endocannabinoids.

In line with the high lipophilicity of THC (Figure 1), all eCBs are obtained from unsaturated fatty acids [4]. The most important of these endogenous ligands are two arachidonic acid (AA)-derivatives: *N*-arachidonoylethanolamine (anandamide, AEA) [5] and 2-arachidonoylglycerol (2-AG) (Figure 1 and Table 1) [6,7], that belong to the large families of *N*-acylethanolamines (NAEs) and 2-monoacylglycerols (MAG), respectively.

**Table 1:** eCBs and eCBs-like compounds, their molecular targets, biosynthetic and catabolic enzymes.

Bioactive Lipids	Molecular Targets	Biosynthetic Enzymes	Catabolic Enzymes
n-6 eCBs derivatives			
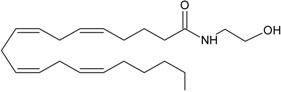 AEA	CB1 [1] CB2 [1] TRPV1 [8] PPARα [9] PPARγ [9] GPR55 [10]	NAT [11] iNAT [12,13,14] NAPE-PLD [15] ABHD4 [16,17,18] Lyso-PLD [16,17,18] GDE1[16,17,18] PTPN22 [16,17,18]	FAAH-1 [19] FAAH-2 [20] NAAH [21] LOXx [22] COX-2 [23,24] CytP450 [25]
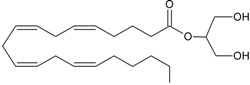 2-AG	CB1 [1] CB2 [1] TRPV1 [26]	PLCβ [27,28] DAGLα [29] DAGLβ [29]	MAGL [30] FAAH-1[19] ABHD6 [31,32] ABHD12 [31,32] LOXx [22] COX-2 [23,24]
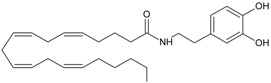 NADA	CB1 [1] TRPV1 [33] PPARγ [34]	Postulate condensation between the catecholamine with AA	Slow hydrolysis of the amide bond or the methylation of catecholamine
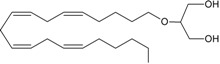 noladin ether	CB1 [35,36] CB2 [35] GPR55 [37] PPARα [9]	unknown	unknown
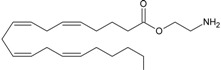 virodhamine	CB1 [38] CB2 [38] GPR55 [37] PPARα [9]	unknown	unknown
n-3 eCBs derivatives			
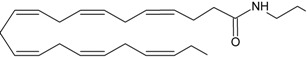 DHEA	CB1 [39] CB2 [39] PPARγ [40]	Postulate as other NAEs	Postulate as other NAEs
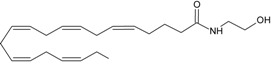 EPEA	CB1 [39] CB2 [39] PPARγ [41]	Postulate as other NAEs	Postulate as other NAEs
**Monounsaturated and saturated fatty acids derivatives**			
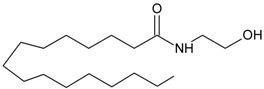 PEA	PPARα [42,43,44,45,46,47,48,49,50,51] GPR55 [52] GPR119 [53]	NAT [11] iNAT [12,13,14] NAPE-PLD [15] Lyso-PLD [16,17,18] GDE1[16,17,18] PTPN22 [16,17,18]	FAAH-1 [54] FAAH-2 [21] NAAH [55]
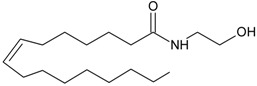 OEA	PPARα [42,43,44,45,46,47,48,49,50,51] GPR119 [53] GPR55[52]	NAT [11] iNAT [12,13,14] NAPE-PLD [15] ABHD4 [16,17,18] Lyso-PLD [16,17,18] GDE1[16,17,18] PTPN22 [16,17,18]	FAAH-1 [54] FAAH-2 [21] NAAH [55]
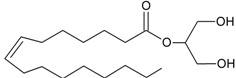 2-OG	GPR119 [53]	PLCβ [27,28] DAGLα [29] DAGLβ [29]	MAGL [30] FAAH-1 [54]

*Notes*: ABHD4, α/β-hydrolase 4; ABHD6/12, α/β-hydrolase domain 6/12; CB_1_, type-1 cannabinoid receptors; CB_2_, type-2 cannabinoid receptors; COX-2, cyclooxygenase-2; Cyt P_450_, cytochrome P_450_; DAGLα/β, diacylglycerol lipase α/β; FAAH, fatty acid amide hydrolase; GPR55, orphan G protein-coupled receptor 55; LOXs, lipoxygenases; MAGL, monoacylglycerol lipase; NAAA, *N*-acylethanolamine-hydrolyzing acid amidase; NAPE-PLD, *N*-acyl-phosphatidylethanolamines-hydrolyzing phospholipase D; NAT, *N*-acyltransferase; iNAT, Ca^2+^-independent *N*-acyltransferase; PLCβ, phospholipase Cβ; PPARα/γ, peroxisome proliferator-activated receptor α/γ; PTPN22, protein tyrosine phosphatase, non-receptor type 22; TRPV1, transient receptor potential vanilloid type 1 channel.

Additionally, other ω-6 (n-6) fatty acid compounds, including *N*-dihomo-γ-linolenoylethanolamine, *N*-arachidonoyldopamine (NADA), 2-arachidonoylglycerylether (noladin ether) and *O*-arachidonoyl- ethanolamine (virodhamine) (Table 1), have also been found to interact with CBRs, though with differing potencies and efficacies as reported in a comprehensive review [1] and summarized in Table 1. Two metabolically important ω-3 (n-3) fatty acid ethanolamines have also been discovered: *N*-eicosapentaenoylethanolamine (EPEA) and *N*-docosahexaenoylethanolamine (DHEA) (Table 1) [56,57]. These n-3 eCBs have been proposed as additional CBRs agonists [58], but their pharmacology and biological relevance remain to be clarified.

The ECS is further complicated by important compounds structurally related to eCBs, and called “eCBs-like” substances. The latter are often found in much higher amounts than AEA, and are devoid of affinity for CBRs; yet, they are metabolized by the same synthesizing and degrading enzymes as authentic eCBs [11]. Among these compounds, the *N*-acyl derivative of palmitic acid and *N*- and *O*-acyl derivatives of oleic acid (Table 1) are particularly relevant.

The biological actions of eCBs and congeners are controlled through not yet fully characterized cellular mechanisms, that include key agents responsible for: (i) AEA and 2-AG synthesis, like the *N*-acyl-phosphatidylethanolamines (NAPE)-hydrolyzing phospholipase D (NAPE-PLD) and the *sn*-1-specific diacylglycerol lipase (DAGL), respectively; and (ii) their degradation, like the fatty acid amide hydrolase (FAAH) and the monoacylglycerol lipase (MAGL), respectively. Remarkably, during the last few years multiple pathways have been described for eCBs metabolism (especially for NAEs), and will be described later in this review.

Strong pharmacological and biochemical evidence has demonstrated that eCBs and related molecules are also able to interact with non-CBR targets, increasing the complexity of the ECS and of the molecular pathways trigged thereof. In particular, the best known non-CBR target of eCBs is the transient receptor potential vanilloid type 1 (TRPV1) channel, which is activated by both AEA [8] and 2-AG [26].

Other potential receptors activated by eCBs are peroxisome proliferator-activated receptor (PPAR) α and γ [9], and the orphan G protein-coupled receptor GPR55 [10,37,52]. In Table 1 old and new members of the ECS are listed together. The ECS has been shown to regulate different physiological processes in the central nervous system (CNS) and at the periphery [59,60,61,62,63], thereby suggesting that its signaling may foster the development of pathway-selective drugs for therapeutic benefit [39,63,64,65,66,67,68].

In this review, we present the state of the art on eCBs, related compounds and their metabolic routes with a mention about their potential therapeutic role.

## 2. Endocannabinoids System

Although many GPCRs have endogenous ligands that are hydrophilic cations, CBRs have neutral, highly lipophilic ligands derived from fatty acids. The differences in reciprocal endogenous concentration of eCBs, receptors affinity, and asymmetrical localization (intracellular and tissutal) of their metabolic enzymes support distinct roles for these molecules under various physiological conditions (e.g., in different forms of synaptic plasticity) [2,69,70,71,72,73,74,75].

### 2.1. Endogenous Ligands of Cannabinoid Receptors

#### 2.1.1. Main Endocannabinoids

The search for endogenous ligands of the THC binding sites has proven difficult at the beginning, because water-soluble substances were searched for by analogy to endorphins. Later on, the lipid nature of THC led to the discovery of two eicosanoids: the *N*-arachidonoylethanolamine, termed “anandamide” from the Sanskrit word “*ananda*” for inner bliss [5], and 2-arachidonoylglycerol (Figure 1) [6,7].

Numerous studies have been carried out on AEA since 1992. This eCB was first found in the brain and then in many other organs and fluids [76]. AEA shares many properties with THC, and acts as a partial agonist of CB_1_ and as a weak partial agonist/antagonist of CB_2_ [1].

However, it should be pointed out that AEA is present, often in low amounts [72], in areas with high or low density of CBRs, suggesting the possibility that it may activate other receptors [1].

The relative low amounts of AEA can be explained by considering the bioavailability of its precursor, and in addition they might suffer from the impossibility to measure accurately their exact local concentration [72].

Instead, the levels of the second eCB, 2-AG, are usually markedly higher than those of AEA in the same tissues [76]. 2-AG acts as a full agonist at CBRs, and has been proposed as the main endogenous agonist for both CB_1_ and CB_2_ receptors [1]. The two main eCBs have different affinity for CBRs, in fact 2-AG is engaged in CB_1_-dependent retrograde signaling, whereas AEA it is only under some condition, causing the inhibition of presynaptic release of the excitatory neurotransmitter glutamate and/or of the inhibitory neurotransmitter GABA [77,78,79].

Notably 2-AG, besides acting as an eCB, is also an important intermediate in lipid metabolism and, therefore, its physiological concentrations may not reflect only the amount needed to trigger CBRs [69]. Indeed, 2-AG has long been regarded as a degradation product of inositol phospholipids, and as a possible source of arachidonic acid in stimulated cells. Moreover, when assessing the levels of 2-AG, it is necessary to keep into account that this compound (much alike all 2-acylglycerols) undergoes acyl migration at room temperature in aqueous media, resulting as an equilibrium of predominant 1(3)-AG (90%) with 2-AG itself (10%) [80].

Furthermore, although different studies support the hypothesis that the composition of dietary fatty acids can affect the levels of eCBs (and eCBs-like compounds) in a time- and tissue-specific manner [56,81,82], yet no changes in blood eCBs were found with low and high-fat diets in obese and normal-weight subjects but, in the same contest, a modification in the skeletal muscle of CB_1_ receptors and MAGL was reported [83]. Other studies have shown that a diet low in n-3 polyunsaturated fatty acids in mice induces a deterioration of CB_1_ receptor functions in the offspring [84]. Notably, the level of eCBs (and eCBs-like compounds) varied depending on the physiological and pathological conditions, and often the content of distinct fatty acid derivatives is regulated independently [38,85] and this different regulation offers the possibility of being able to selectively act on the concentration of the derivative of interest with a potential beneficial effect.

#### 2.1.2. Additional n-6-Endocannabinoids

During the past years, different AA derivatives with cannabimimetic properties have been detected, suggesting the existence of new members of the endocannabinoid family. In particular, an ether-type eCB, 2-arachidonoyl-glyceryl ether or noladin ether (Table 1) [84], and an AA and ethanolamine derivative with an ester bond (an “inverted” AEA), called virodhamine (Table 1) [36], have been isolated in the brain. Additionally, *N*-arachidonoyldopamine (NADA), that is primarily a TRPV1 agonist, has some activity at CB_1_ as well (Table 1) [86]. These compounds have received less attention than the two main eCBs (AEA and 2-AG), maybe due to the difficulty for many researchers to isolate them from biological tissues [72,87].

At any rate, it has been reported that noladin ether binds to CB_1_ receptors and very weakly to CB_2_ receptors [35], besides affecting AEA uptake [88]. Moreover, virodhamine has been shown to behave *in vitro* as a CB_2_ receptor full agonist, and as a partial agonist of CB_1_. Instead, *in vivo* it is an antagonist at CB_1_, and also a weak inhibitor of AEA uptake [36].

Much like AEA, noladin ether and virodhamine interact, although with different affinity, with PPARα and the orphan GPR55 receptor [9,37,87,88,89,90,91], again suggesting that non-CBRs can be common targets for several fatty acid derivatives.

The last derivative of AA which has been added to the eCBs family has been so far is NADA [86], which shares with AEA, and with its analogue *N*-oleoyldopamine [33], the ability to activate TRPV1. Indeed, NADA has been found in bovine brain areas with elevated density of TRPV1 channels, and is considered a true “endovanilloid” [92]. Furthermore, AEA and NADA seem to share also PPARγ as a target [34]. NADA has been found in brain regions with the highest amounts of dopamine, thus it was suggested to be the product of condensation of this catecholamine with AA [86,93]. Conversely, its inactivation likely goes through a very slow hydrolysis of the amide bond, or through the methylation of the catecholamine moiety by catechol-*O*-methyl transferase, with the formation of a less potent 3-*O*-methyl derivative [86].

Besides AEA, other less known ω-6 (n-6) unsaturated NAEs, able to interact with CBRs and endowed with three or four double bonds, are also formed, apparently even in higher amounts than AEA. Among these, the *N*-dihomo-γ-linolenoylethanolamine has been isolated from different tissues [94,95], as well as from biological fluids [40], where it acts as a weak CBRs agonist, yet with a poorly understood (if any) physiological significance.

#### 2.1.3. n-3-Endocannabinoids

Since the 1930s essential roles were assigned to the n-3 polyunsaturated fatty acids (PUFAs), because their lack in the diet gave rise to not yet known forms of deficiency diseases at the time [96]. There is a lot of literature that speaks of the various beneficial effects of PUFAs such as protective effects against cardiovascular disease, inflammation, and cancer [97,98], although there are now several articles that challenge the beneficial effects of these fatty acids [99].

Interestingly, among the molecules capable of activating CBRs metabolites derived from PUFAs were detected. In particular, two derivatives of docosahexaenoic acid (DHA, C22:6) and eicosapentaenoic acid (EPA, C20:5) were found, and were called DHEA and EPEA respectively (Table 1) [39]. Even these n-3 NAEs showed the same promiscuity of the corresponding n-6 analogues, and indeed in addition to bind to CB receptors they are also able to activate PPARγ [41].

Furthermore, n-3 PUFAs and their natural derivatives resolvins can modulate TRPV1 activity [100,101,102]. In line with this, the bioactive derivative of DHA, resolvin D2, inhibits TRPV1 currents in dorsal root ganglion neurons, although the underlying mechanism does not seem to be direct, but rather mediated by an unknown GPCR [101]. The ability of resolvins to reduce inflammation under physiological conditions [103] makes these molecules very attractive as possible anti-inflammatory/analgesic drugs.

Altogether, endocannabinoid signaling appears rather complex, and seems to be clearly affected by diet (with particular reference to the n-3/n-6 ratio). For instance, DHEA can be found under basal conditions, whereas EPEA is detected in the same cells only when supplied with an appropriate diet [42,57]. These n-3 eCBs have shown anti-inflammatory properties in macrophages [43] and adipocytes [3], and can inhibit cell growth in breast cancer by triggering autophagy via PPARγ [41]. Remarkably, oral administration of DHEA has been shown to have beneficial effects in patients who were poor responders to *in vitro* fertilization treatments [45].

#### 2.1.4. Endocannabinoid-Like Compounds

AEA belongs to a class of naturally occurring molecules (NAEs) known for a long time. One of its members, *N*-palmitoylethanolamine (PEA), was first reported almost 50 years ago in humans, yet its physiological relevant remains under debate when the mechanism is other than via CBRs [46].

PEA and other NAEs share with true eCBs many degradative mechanisms, and they potentiate the effect of eCBs at their receptor targets by competitively inhibiting their hydrolysis, or by allosterically modulating their receptor binding: the so-called “entourage effect” [47,48]. On this basis, these substances are also known as “eCBs-like” compounds (Table 1).

Among the most studied eCBs-like compounds, the anti-inflammatory agent PEA and the appetite-suppressor *N*-oleoylethanolamine (OEA) can be listed (Table 1) [46]. Their biological activity often engages PPARα and TRPV1 activation [47,48,49,50,51], although some of their actions are prevented by CB_1_ antagonists [104,105]. Among the eCBs-like compounds OEA shows the highest affinity for PPARα [106], and consistently some of its biological effects are absent in PPARα deficient mice [107]. Yet, the antinociceptive properties of OEA are exercised also through a PPARα-independent mechanism [108].

OEA, as well as PEA and 2-oleoylglycerol (2-OG) (Table 1), can also activate GPR119, a GPCR expressed predominantly in human and rat pancreas [53], suggesting that the effects of OEA on food intake may be mediated, at least in part, via GPR119 [53,109,110]. Conversely, Lan and coworkers reported that the hypophagic effect of OEA was preserved in Gpr119(-/-) mice [111]. Not surprisingly, there is also evidence that OEA (as well as PEA) can engage, even though at high concentrations, additional receptors like GPR55 [52].

Another saturated NAE, *N*-stearoylethanolamine (SEA), was reported to act as a cell growth controller and anti-inflammatory/immunomodulatory agent, through yet unknown targets [46,112,113]. SEA also shows anorexic effects that are PPAR-independent [114] and, together with PEA, plays an antinociceptive role in humans [114].

As reported above, also the endogenous levels of these eCBs-like compounds (PEA, OEA and SEA) are affected by different dietary regimens, with different hits in the brain compared to peripheral tissues [56,82].

**Figure 2:**
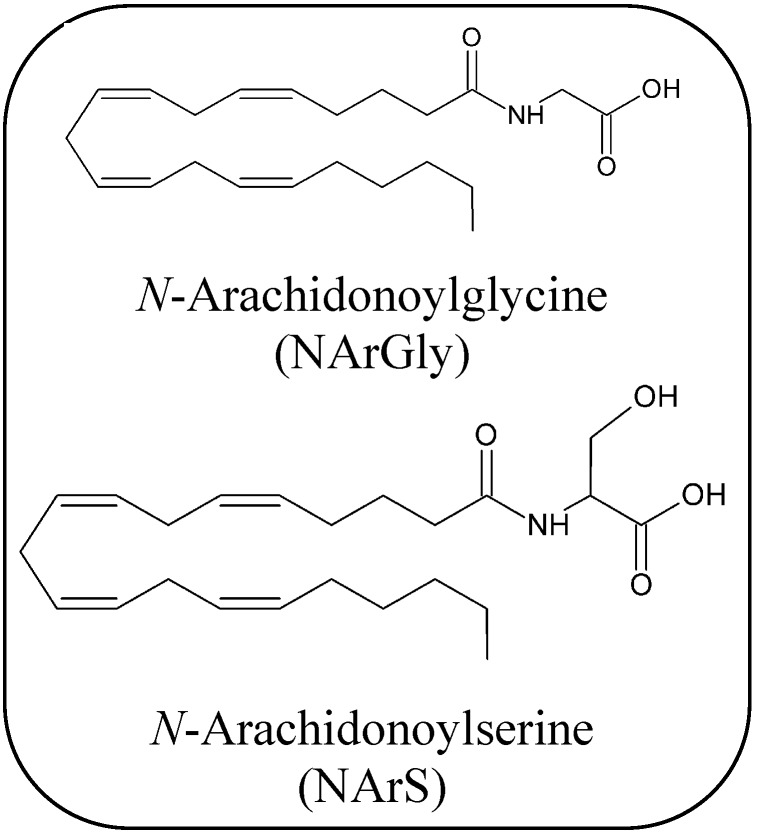
Chemical structures of some endocannabinoid-like molecules.

Recent advancement of analytical techniques has allowed to detect a large variety of compounds containing fatty acid chains conjugated with different polar heads [87,115,116]. Within these novel lipids, *N*-arachidonoylglycine (NArGly) and *N*-arachidonoylserine (NArS) (Figure 2) as arachidonoyl-amino acids can be listed [53,109,110,111,112,113,114,115,116,117,118,119,120].

NAGly, that differs from AEA only for the oxidation of the β carbon, acts as a high affinity ligand for GPR18 [121,122,123], and as a partial agonist of Gq/11−coupled GPR92 receptors [124]. NAGly was first showed to be a potent *in vitro* FAAH inhibitor [125], and later on it was shown to occur naturally *in vivo*, to mimic the pharmacological profile of abnormal cannabidiol [119], and to exert an indirect neuroprotective effect through CB_2_ and TRPV1, but not CB_1_ or GPR55, receptors [126].

Although further investigations are necessary to elucidate the actual physiological relevance of eCBs-like compounds and arachidonoyl-amino acids, it is important to point out that PEA, due to the lack of adverse side-effects at central CB_1_ receptors holds potential for the development of innovative medicine, and it is currently marketed to cure neuropathic and pelvic pain [127].

### 2.2. Metabolism of Endocannabinoids and Related Compounds

Much like many other bioactive molecules, the activity of eCBs is controlled by their endogenous levels, and therefore by a balance between biosynthetic and degradative mechanisms. Based on original observations carried out on AEA [128], it was postulated that eCBs are not stored in pre-formed vesicles, yet they are rather synthesized and released “on demand”, *i.e.*, when and where needed. However, more recent views have imposed a reconsideration of this “dogma”, because AEA can be stored in lipid droplets (adiposomes) and is bound to intracellular transporters [2,129]. A modern view of the metabolic pathways of AEA and 2-AG (and related substances) is presented in the next sections.

#### 2.2.1. Biosynthesis of AEA and Congeners

**Scheme 1:**
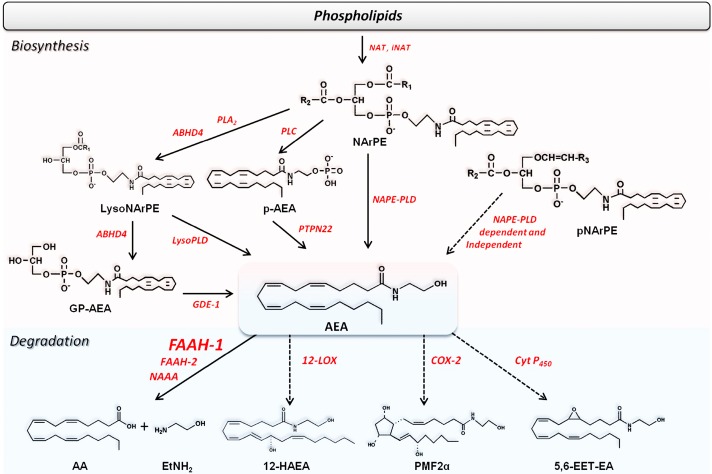
The alternative biosynthetic and degradative pathways of AEA and congeners.

*Notes*: NAAA, *N*-acylethanolamine-hydrolyzing acid amidase; NArPE, *N*-arachidonoyl- phosphatidylethanolamine; pNArPE, *N*-arachidonoylethanolamine plasmalogen; ABHD4, α/β-hydrolase 4; NAPE-PLD, *N*-acyl-phosphatidylethanolamines-hydrolyzing phospholipase D; AA, arachidonic acid; COX-2, cyclooxygenase-2; Cyt P_450_, cytochrome P_450_; EET-EA, epoxyeicosatrienoyl ethanolamides; EtNH_2_, ethanolamine; FAAH, fatty acid amide hydrolase; GP-AEA, glycerophospho-AEA; GDE1, glycerophosphodiester phosphodiesterase 1; 12-HAEA, 12-hydroxyanandamide; *lyso*-NArPE, lyso-*N*-arachidonoylphosphatidylethanolamine; 12-LOX, 12-lipoxygenase; pAEA, phospho-AEA ; NAT, *N*-acyl-transferase; iNAT, Ca^2+^-independent *N*-acyltransferase; PMF2α, prostamides F2α; PLA_2_, phospholipase A_2_; PLC, phospholipase C; *lyso*-PLD, *lyso*-phospholipase D; PTPN22, protein tyrosine phosphatase, non-receptor type 22.

The ever-growing number of enzymes involved in the biosynthesis of AEA (and related NAEs) suggests that the endogenous tone of this eCB is subjected to a highly complex and highly regulated network of reactions (Scheme 1).

The main route for NAEs biosynthesis consists of two enzymatic reactions. The first is a fatty acyl chain transfer from membrane phospholipids to a phosphatidylethanolamine, resulting in the formation of *N*-acylphosphatidylethanolamine (NAPE), by a yet-unidentified Ca^2+^-dependent *N*-acyltransferase (NAT) [11] or a Ca^2+^-independent counterpart (iNAT) [12,13,14]. Being the palmitoyl and oleoyl acids preferentially incorporated in *sn*-1 position, NAT preferably produces PEA and OEA rather than AEA [11]. Instead, iNAT removes a fatty acyl group from both the *sn*-1 position and the *sn*-2 position (where AA is most abundant) of phosphatidylcholine (PC), that is the acyl donor [13,14].

Furthermore, NAT and iNAT have different cellular and tissue localizations, for example the latter is poorly expressed in the brain [11] suggesting a distinct role in the control of NAEs levels.

The second step is catalyzed by a type D phospholipase (NAPE-PLD) (Scheme 1), that is distinct from the classical PLDs. Indeed NAPE-PLD, that is highly conserved from rodents to human belongs to the metallo-lactamase family of enzymes, and *in vitro* it catalyzes the formation of AEA from its C20:4-NAPE precursor, as well as by other NAPEs [15].

Accumulated evidence indicates, however, the existence of additional pathways for AEA formation from NArPE [11,130]. Indeed, through the use of knock-out mice several enzymes and metabolites involved in the NAPE-PLD-independent biosynthesis of AEA have been identified and characterized [16,17,18]. These alternative pathways of AEA are shown in Scheme 1. More recently, an interesting paper reported a novel route for NAEs formation from *N*-acylethanolamine plasmalogen (1-alkenyl-2-acyl-glycero-3-phospho(*N*-acyl)ethanolamine, pNAPE), one of the major classes of glycerophospholipids in mouse brain [131]. Also this route is depicted in Scheme 1.

#### 2.2.2. Biosynthesis of 2-AG and Congeners

**Scheme 2:**
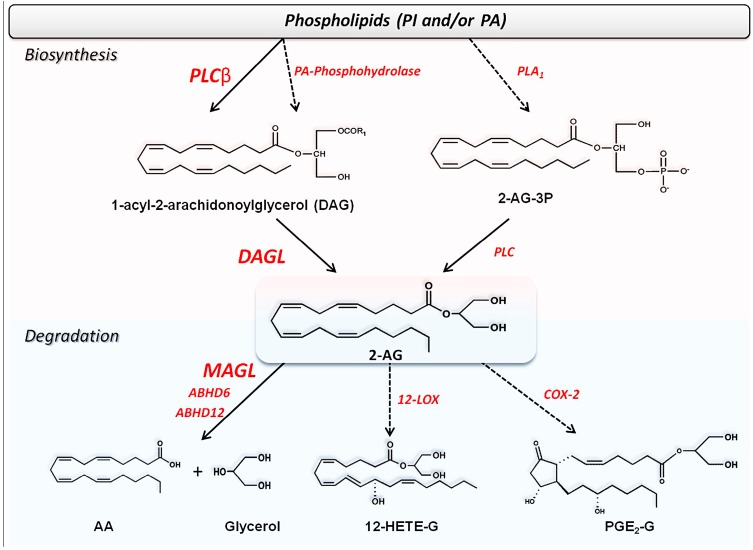
The alternative biosynthetic and degradative pathways of 2-AG and congeners.

*Notes*: AA, arachidonic acid; 2-AG-3P, 2-arachidonoylglycerol-3-phosphate; COX-2, cyclooxygenase-2; DAG, diacylglycerol; DAGL, diacylglycerol lipase; 12-HETE-G, 12-hydroxyarachidonoyl-glycerol; ABHD6/12, α/β-hydrolase domain 6/12; 12-LOX, 12-lipoxygenase; MAGL, monoacylglycerol lipase; PLC, phospholipase C; PLCβ, phospholipase Cβ; PGE_2_-G, prostaglandinglycerol E_2_-G.

The best known biosynthetic pathway for 2-AG requires the combined action of two membrane enzymes: phospholipase C (PLC) and diacylglycerol lipase (DAGL), as shown in Scheme 2. In particular, between various PLC isoforms [132], β1 and β4 have been linked to 2-AG formation triggered by GPCRs in hippocampal neurons and cerebellar Purkinje cells, respectively [27,28]. Moreover, since PLC recognizes different phospholipids, the distribution of which varies among tissues, formation of DAG, and then of 2-AG, by such a lipase is tissue-specific [133,134]. Alternative pathways of 2-AG biosynthesis are shown in Scheme 2.

DAGL, the second enzyme involved in 2-AG formation, is present in two forms, α and β [29]. Thus, the high content of AA at the latter position can explain the predominant production of 2-AG over other MAGs [29]. Remarkably, two independent studies have suggested that only 2-AG generated by DAGLα is responsible for retrograde suppression through CB_1_ at central synapses in the brain [135,136]. Furthermore, decreased AEA levels in the cerebellum and hippocampus of daglα-/- mice have been reported [136], supporting a mutual interaction between AEA and 2-AG, possibly engaging also TRPV1 channels as documented in the striatum [137]. Additionally, a regulation of DAGLα by calcium/calmodulin-dependent protein kinase II was reported [138], and such a protein kinase had been previously shown to regulate also TRPV1 receptor [139]. The alternative pathways of 2-AG biosynthesis are shown in Scheme 2.

#### 2.2.3. Degradation of Endocannabinoids and Congeners

##### Uptake of Endocannabinoids and Congeners

At the beginning the mechanism involved in eCBs transmembrane transport that received most attention was facilitated transport [140,141,142], yet no transporter protein has been yet cloned. Then, additional mechanisms have been proposed, that have been recently reviewed [143]: (i) passive diffusion gated by FAAH [144,145], by intracellular sequestration [146], or by the formation of AEA-cholesterol complexes [147]; (ii) caveolae-dependent endocytosis [143].

Another hot topic is the understanding of how eCBs can reach their distinct sites of action within the cell (e.g., membrane or nuclear receptors, or metabolic enzymes) at the right time, in order to trigger the appropriate response to a stimulus. In this context, the existence of intracellular storage organelles (lipid droplets or adiposomes) [146], and of constitutive intracellular transporters (AEA intracellular transporters, AITs) have been reported for AEA. Among constitutive AITs are heat shock protein 70 (Hsp70) and albumin [148], to which fatty acid binding proteins 5 and 7 (FABP5 and FABP7) have been added as exogenous entities [149]. Interestingly, a functional role for FABPs in endocannabinoid signaling has been recently documented [150], providing a proof of concept that indeed AITs can drive endocannabinoid signaling through a distinct pathway (e.g., the one triggered *via* PPARα by OEA [150].

Moreover, it has been suggested that some NAEs are accumulated in cells via a general mechanism shared with AEA [151], which can operate in a bi-directional mode [141,151,152]. Interestingly, PEA has been shown to interfere with AEA transmembrane transport in a cell type-dependent manner [153], and several analogs of OEA were shown to be more powerful than NAEs containing AA in blocking AEA uptake [128]. Less information is available on the cellular accumulation of other eCBs analogues, yet at least one double bond in the acyl chain seems necessary for the transmembrane transport to take place [143,154].

As yet, only a few studies have addressed the transport of 2-AG, but apparently this eCB uses the same mechanism used by AEA [151,155,156]. In addition, 2-AG can be directly esterified into (phospho)glycerides, via phosphorylation and/or acylation of its free hydroxyl groups [156].

In conclusion, the following citation appears quite instructive: “Translocation across the plasma membrane is achieved by a concert of co-existing mechanisms. These lipids can passively diffuse, but transport can also be accelerated by certain membrane proteins as well as lipid rafts” [157].

##### Hydrolysis of AEA and Congeners

Probably the main catabolic enzyme responsible for signal termination of AEA is fatty acid amide hydrolase (FAAH) [19]. This enzyme has been cloned by Cravatt and coworkers in 1996 [35,54], and shown to break down AEA, as well as other NAEs and also 2-AG (Table 1 and Scheme 1).

FAAH is an intracellular membrane-bound serine hydrolase with S241-S217-K142 as catalytic triad [158,159]. This hydrolase is widely present in the brain, where it shows a subcellular distribution that overlaps on that of CB_1_ receptors, and in virtually all peripheral organs, yet with a different distribution between rodents and humans [158].

A few years after the characterization of FAAH (now called FAAH-1), two other hydrolases able to hydrolyze AEA, were reported and recently revisited [55]: an isoform of FAAH that is known as FAAH-2 [20], and a lysosomal cysteine hydrolase termed *N*-acylethanolamine-hydrolyzing acid amidase (NAAA) (Scheme 1) [21]. In particular, FAAH-2 has a limited species distribution in mammals, and appears to be permanently-associated with adiposomes [160], where AEA can be stored, and was localized also on lipid droplets [146]. FAAH-1 and FAAH-2 share limited sequence identity (~20%), while no homology exists between them and NAAA [21], an enzyme that shows a substrate preference toward other saturated or monounsaturated NAEs [55]. Interestingly, several potent NAAA inhibitors have been shown to potentiate the effect of PEA at PPARα [161,162]. Instead, the primary role of FAAH-1 appears to control the *in vivo* levels of AEA and other polyunsaturated NAEs, as confirmed by FAAH1-/- mice [163].

##### Hydrolysis of 2-AG and Congeners

Solid evidence demonstrates that monoacylglycerol lipase (MAGL) is the main responsible for 2-AG degradation *in vivo* (Table 1 and Scheme 2). Indeed, hydrolysis and content of 2-AG remain unchanged in FAAH1-/- mice, while they markedly increased in MAGL-/- mice [30], and were associated with profound changes in 2-AG signaling [164]. Furthermore, the different localization of MAGL and FAAH-1 in the brain [30,70] supports the hypothesis of distinct roles for these two eCBs [165].

Interestingly, MAGL exhibits higher specificity than FAAH-1, because treatment with its potent and selective inhibitor JZL-184 rises only the levels of 2-AG, without affecting those of any other MAG (e.g., monopalmitoylglycerol and monooleoylglycerol) [166]. MAGL is a serine hydrolase with a catalytic triad (S122-D239-H269) that is highly conserved among different species [166,167,168,169,170]. Interestingly, different observations support a role for MAGL as a provider of free fatty acids, that sustains cancer [171,172,173].

Two additional serine hydrolases, α/β-hydrolase domain 6 (ABHD6, with a postulated catalytic triad S148-D278-H306) and 12 (ABHD12, with a postulated catalytic triad S246-D333-H372), are involved in 2-AG hydrolysis (Table 1 and Scheme 2) [31,32]. Of note, MAGL, ABHD6 and ABHD12 show a distinct distribution within the CNS [32,174], that is suggestive of a different physiological function of these three enzymes in regulating 2-AG signaling [32,175]. In support of this view, anti-inflammatory effects of ABHD6 inhibition without the side effects typically associated with MAGL inhibition have been recently reported [176].

Furthermore, mutations in *abhd12* gene are associated with the neurodegenerative disease called PHARC (polyneuropathy, hearing loss, ataxia, retinitis pigmentosa, and cataract) [177]. It could speculate that this inherited disease can be linked to a dysfunction of the 2-AG metabolism, although a recent study on ABHD12^−/^^−^ mice would seem to involve the ECS in this disease [178].

##### Oxidative Metabolism of eCBs and eCBs-Like Compounds

Alternatively to hydrolytic routes, eCBs are also substrates of the enzymes that oxygenate AA. These catabolic pathways represent an interesting point of intersection between endocannabinoid and classical eicosanoid systems, leading to the production of new biologically active metabolites [23].

**Figure 3:**
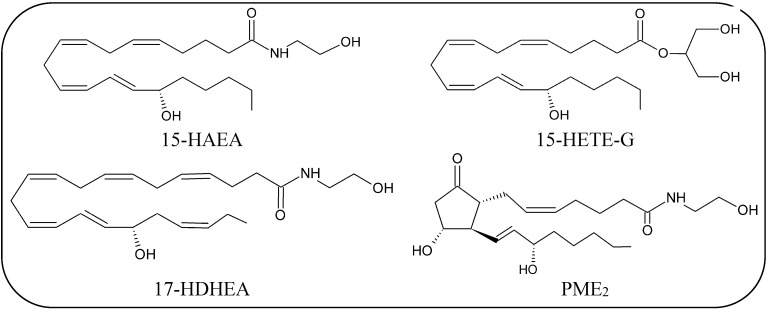
Chemical structures of the main products of oxidative metabolism of AEA and 2-AG.

In particular, AEA and 2-AG are metabolized by lipoxygenases (LOXs) [22] and by cyclooxygenase-2 (COX-2) [23,24], and additionally AEA can be oxygenated also by cytochrome P_450_ (Table 1). [25]. The main products of the oxidative metabolism of AEA and 2-AG are reported in Scheme 1, Scheme 2 and Figure 3.

Several pharmacological data point to LOX-derivatives of AEA (hydroxyanandamides, HAEAs) as ligands for CB_1_, CB_2_, PPARs, and TRPV1 receptors [179,180,181], and they can also interact with some enzymes of the ECB system [22,182], as well as with cell membranes properties [183,184].

In addition to AEA, other fatty acid derivatives like DHEA, NArGly and *N*-arachidonoyltaurine undergo LOX-catalyzed oxygenation [185,186,187]. In particular, 17-hydroxy-DHEA (Figure 3) occurs naturally in mouse brain, where it appears to exert a protective function [185].

COX-2, but not COX-1, can oxygenate the main eCBs generating a number of oxygenated molecules [23]. By analogy with prostanoids, the products of AA oxygenation by COX-2, AEA and 2-AG can be oxygenated to leads to prostaglandin ethanolamides (prostamides, PMs) and prostaglandin glycerol esters (PGs-G), respectively (Scheme 1, Scheme 2 and Figure 3) [188]. Unlike LOX-generated derivatives of eCBs, the biological activities of COX-2-derived metabolites are probably mediated by distinct receptors compared to those that bind eCBs [23,183,189]. Interestingly, a number of weak inhibitors of AA oxygenation by COX-2, like nonsteroidal anti-inflammatory drugs (NSAIDs), are potent inhibitors of endocannabinoid oxygenation by the same enzyme [190,191], suggesting that NSAIDs may be useful to better understand the pharmacological properties of PMs *in vivo*. Interestingly, an increase of prostamide F_2α_ (PMF_2α_) was found in the spinal cord in mice, after induction of inflammation, and was found to exert a proalgesic effect, supporting the relevance of endocannabinoid oxidation *in vivo* [192]. Furthermore, the PMF_2α_ analogue bimatoprost is currently used for the treatment of glaucoma [193]. More recently, prostaglandin D_2_-glycerol ester was found to decrease macrophage activation, and this effect was dependent on ABDH6 activity [176].

Finally, AEA can be metabolized by several different human cytochromes P_450_, to form a number of structurally related epoxyeicosatrienoic ethanolamides (EETs-EA) [185]. In particular, AEA epoxide at positions C5–C6 (5,6-EET-EA) (Scheme 1) is generated by human CYP3A4, an isoform of cytochrome P_450_, and acts as a potent and selective agonist of CB_2_ receptors [194]. Instead, the orphan cytochrome P_450_ 4X1 was found to produce 14,15-EET-EA, whose (patho)physiological relevance remains to be clarified [195].

## 3. Conclusions

The ubiquity of eCBs and their multiple (patho)physiological implications has allowed to identify novel targets for next generation therapeutics; yet, the numerous side effects at the central and peripheral levels may dampen enthusiasm towards these new targets. Indeed, recently they led to the withdrawal of Acomplia^®^ (rimonabant), a CB_1_ receptor antagonist/inverse agonist that was licensed and marketed as an anti-obesity agent [67].

However, over the last 20 years eCBs-related drugs have indeed been commercially available, such as Cesamet^®^ (nabilone, a synthetic cannabinoid) and Marinol^®^ (dronabinol, a synthetic THC), that are preparations used to treat nausea and vomiting associated to cancer chemotherapy. The latter drug is also prescribed to manage the loss of appetite in people with acquired immunodeficiency syndrome (AIDS). In addition Sativex®, a medicine that contains THC and cannabidiol at a 1:1 ratio, has been licensed for the symptomatic treatment (pain, spasticity and incontinence) of multiple sclerosis patients in a (ever-growing) number of countries (e.g., United Kingdom, Canada, New Zealand and United States of America). Furthermore, PEA is currently marketed to cure neuropathic (Normast^®^) and pelvic (Pelvilen^®^) pain, and is one of the main components of a cream (Physiogel^®^) used for inflamed or irritated skin of subjects with atopic dermatitis.

To this aim, modulators of endocannabinoid metabolic routes have been tested, with a promise to be free of unwanted side effects typical of compounds that activate eCBs-binding receptors. These studies have led to opposite results, the FAAH blockade did not lead to desensitization of CB_1_ receptors [196], while the chronic pharmacological inactivation of MAGL causes alterations in CB_1_ receptor function [197]. In conclusion, the development of eCBs-based drugs with a restricted target area appears very promising to cure or slow down different human pathologies.
